# The Role for Preperitoneal Pelvic Packing in Low-to-Middle-Income Countries: A 16-Year Experience at a Johannesburg Trauma Unit

**DOI:** 10.1007/s00268-023-07173-4

**Published:** 2023-09-17

**Authors:** Maeyane Stephens Moeng, Francois Viljoen, Shumani Makhadi

**Affiliations:** Department of Surgery, School of Clinical Medicine, Faculty of Health Sciences, University of the Witwatersrand, Johannesburg, 2193 South Africa

## Abstract

**Introduction:**

Preperitoneal pelvic packing for early pelvic haemorrhage control reduces mortality. Bleeding noted with pelvis fractures is predominantly due to associated venous complex injuries. More studies are advocating for angiography as first-line therapy for haemodynamic instability in pelvic fractures; however, these facilities are not in abundance in middle- and low-income countries. We hypothesized that PPP improves outcomes under these circumstances.

**Methods:**

Retrospective analysis of data from the patients charts over a period of 16 years from 01 January, 2005 to 31 December, 2020. All patients over the age of 18 years who presented with haemodynamic instability from a pelvic fracture and required PPP were included. The demographics, physiological parameter in emergency department, blood products transfused, morbidity and mortality were analysed.

**Results:**

There were 110 patients identified in the study period who underwent pelvic preperitoneal packing for refractory shock or ongoing bleeding. The majority (75.5%) of patients were men (*n* = 83). The median age was 38 years. The most common mechanism of injury was pedestrian vehicle collision (51%), followed by motor vehicle collisions (27.3%). The median ISS and NISS were 35 and 40, respectively. The median RTS in ED was 4.8(3–6.8). None of our patients rebleed after pack removal and no one needed repacking or adjunct angioembolization in our study group. The in-hospital mortality rate was 43.6% (*n* = 48) in patients who underwent preperitoneal pelvic packing. The operating room table mortality was 20% (*n* = 22/110), and the mortality rate of those who survived to ICU transfer was 29.5% (*n = *26/88).

**Conclusions:**

Pelvic preperitoneal packing has a role in the acute management of haemodynamically abnormal patients with pelvic fractures in our environment. In the absence of immediate angioembolization, preperitoneal packing can be lifesaving.

## Introduction

Unstable pelvic fracturs typically occur because of high energy injuries and can be life-threatening [1]. Most pelvic fractures are stable, and only a subset present with haemodynamic instability. Demetriades et al. have shown that unstable pelvic fractures constitute up to 3% of all fractures in blunt trauma [2]. The haemodynamic instability can also be secondary to associated injuries, and in some instances, due to non-haemorrhagic causes like severe head injuries. Mortality rate ranges from 6 to 35% [3, 4]. Mortality in pelvic fractures associated with haemodynamic instability is worse than in stable patients [3, 4].

Preperitoneal pelvic packing for early haemorrhage control reduces mortality [3, 4]. Bleeding noted with pelvis fractures is predominantly due to associated venous complex injuries in about 90% of the cases [3, 4]. However, in haemodynamically unstable fractured pelvis, the arterial component can be as high as 30% [4–6]. Massive transfusion with appropriate blood components has been shown to be beneficial in acute trauma bleeding [7, 8]. This is complemented by evaluating lactate level and base excess to optimize resuscitation [7, 8].

Early stabilization of the fractured bone with binders or a sheet and possible fixation with an external fixator or C-clamp helps reduce further bleeding [9–13]. The high percentage of arterial bleeding in the pelvis supports the need to use angioembolization as part of the strategy to stop the bleeding. Angioembolization is not always easily accessible. The time to completion of angioembolization is also variable [14, 15]. Multiple attempts may be required to achieve satisfactory occlusion of the bleeding vessel [14, 15]. Unfortunately, not all trauma units have access to the angioembolization facilities 24 h-a-day to control the associated arterial bleeding noted in fractured pelvis [16–19].

Preperitoneal pelvic packing (PPP) has been demonstrated to improve outcomes and reduce mortality in bleeding pelvic fractures [20–25]. Abdominal swabs are used to pack the preperitoneal space, with most of the dissection done by the haematoma in the area. When packing, care must exercise at to not damage the sacral plexus. PPP is associated with septic complications like sepsis, especially when packs are left for too long [26].

Haemodynamic unstable patients with pelvic or suspected pelvic fractures who present to the Charlotte Maxeke Johannesburg Academic hospital would have a pelvic sheet at the level of the greater trochanters applied. Fluid resuscitation is initiated with early blood product transfusion. An extended focused assessment with sonography (E-FAST) and computer tomography (CT) were done when indicated [27–29]. Non-responders and transient responders are taken for laparotomy and preperitoneal packing as soon as possible/feasible. The laparotomy facilitates the identification of associated hollow viscus injuries. The preperitoneal pelvic packing is then done, and in some cases, an orthopaedic consult for an external fixator is warranted. Patients would then be admitted to ICU for ongoing resuscitation and supportive care. Once resuscitated in 48–72 h, patients would be taken back for removal of packs.

We do not always have angioembolization readily available at our institution. We, therefore, still do preperitoneal pelvic packing (PPP) for patients with pelvic fractures who present with haemodynamic instability in our unit and fail to improve on fluid resuscitation or deteriorate despite adequate fluid and blood products resuscitation. We considered blood pressure less than 90 to represent haemodynamic instability. We wanted to audit the role of preperitoneal pelvic packing in these circumstances. We hypothesized that PPP improves outcomes in our environment where there is no easy access to angioembolization.

## Aim of the study

We wanted to evaluate the indications and outcomes of PPP in trauma patients with haemodynamic instability presenting at the Trauma Unit in Johannesburg.

## Method

A retrospective cohort study of all fractured pelvis patients who presented with haemodynamic instability at Charlotte Maxeke Johannesburg Academic Hospital (CMJAH) Trauma unit from 01 January, 2005 to 31 December, 2020. Patients 18 years and older were included in the study. Those with missing records were excluded from the study.

Data were collected from the emergency department (ED) records. Clinical records and mortality and morbidity records. The data collected included demographics, physiological status in ED (blood pressure (BP), pulse rate, respiratory rate, Glasgow Coma Scale (GCS), the Base excess, lactate levels, Revised Trauma Score (RTS) in ED and Transfusion requirements during the first 48 h of stay. The Injury Severity Score (ISS) and the New Injury Severity Score (NISS) were also documented. The pelvic fractures anatomy and associated injuries were recorded. The PPP-related surgical complications and in-hospital mortality were also documented. In case of in-hospital mortality, the determination of the cause of the mortality was noted.

## Data were anonymized and documented on the Excel spreadsheet for further analysis

Means (standard deviations, SD) and medians (interquartile ranges, IQR) were calculated for normally distributed and skewed continuous variables, respectively. Frequencies and percentages were used to describe distributions of categorical variables. All analyses were done using STATA version 15. Continuous variables were first tested for normality using the Shapiro–Wilk test. Fisher’s exact test was used to test the significance of the relationship between categorical variables. A *p-value* of < 0.05 was considered statistically significant.

Logistic regression was used to find the relationship between death and explanatory variables. Variables used in our regression model were selected based on their importance in literature and according to the *p*-value from univariate analysis, where we selected variables with *p* ≤ 0.10. We ran both univariate and multivariate logistic regression models. In univariate logistic regression, we obtained crude estimates. In the multivariate models, we adjusted for age, sex and mechanism of injury and obtained adjusted estimates. Odds ratios (OR) and corresponding 95% confidence were used to measure the size of the effect.

## Results

**Fig. 1:**
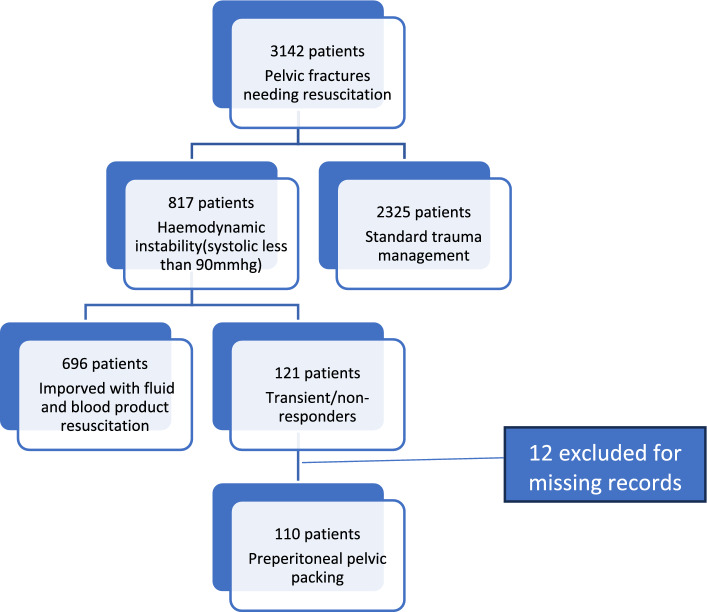
Flow diagram

**Fig. 2:**
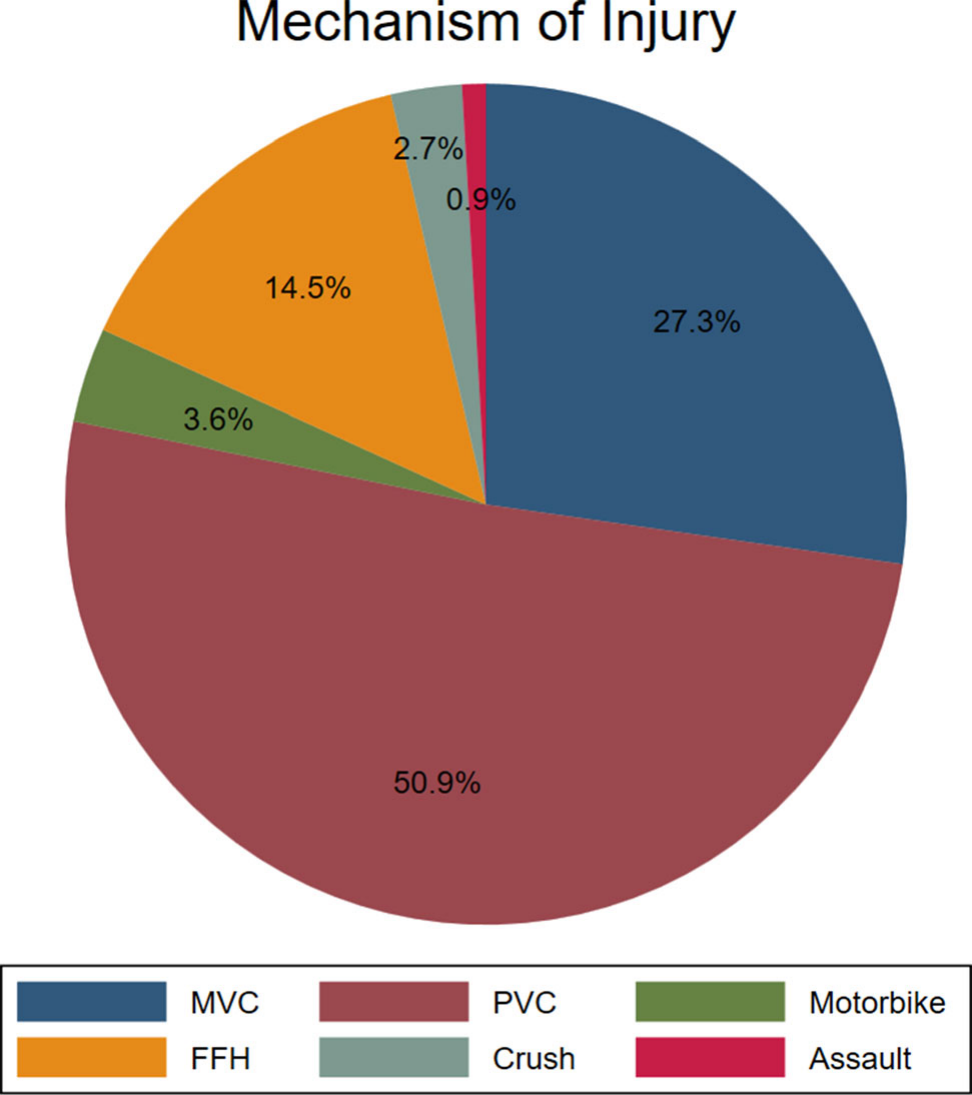
Mechanism of injury

There was a total of 3142 patients with pelvic fractures during the study period. There were 110 patients identified in the study period who underwent pelvic preperitoneal packing for refractory shock or ongoing bleeding (Fig. 1). The majority (75.5%) of patients were men (*n* = 83). The median age was 38 years. The most common mechanism of injury was pedestrian vehicle collision (51%), followed by motor vehicle collisions (27.3%) (Fig. 2).

**Fig. 3:**
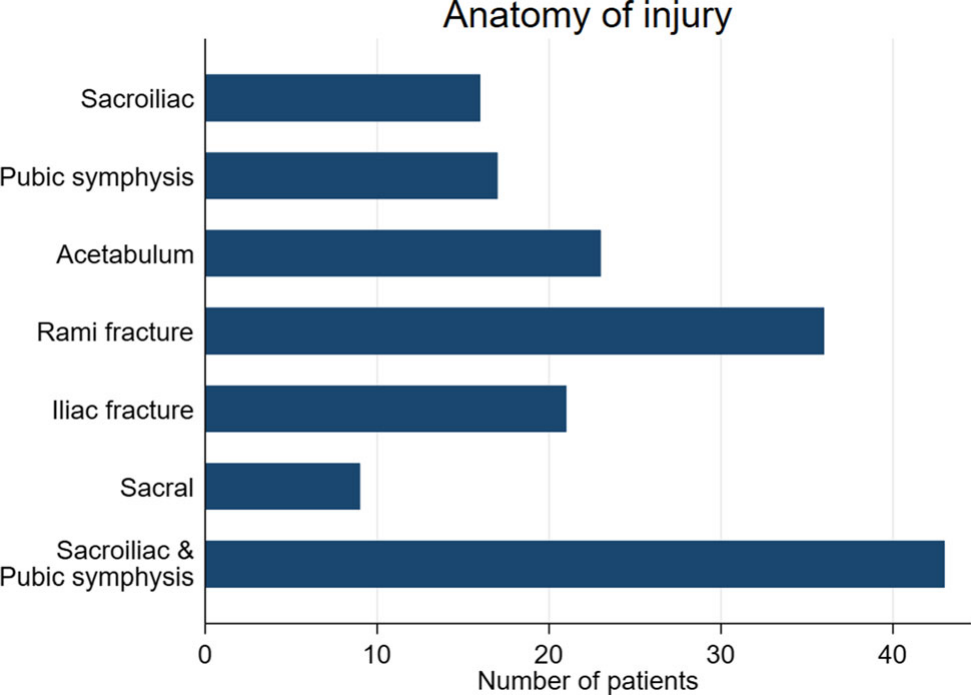
Pelvic fracture patterns

The median ISS and NISS were 35 and 40, respectively. The median RTS in ED was 4.8 (3–6.8). The average pelvic AIS was 4 (Table 1). The pelvic fracture anatomy included combined sacroiliac and pubic symphysis fractures (39.1%), pubic rami fracture (32.7), ilium fracture (19.1%) and sacral fractures (8.2%) (Fig. 3). The mean blood pressure in ED was 87 mmHg, with 22 patients (21.1%) presenting with systolic blood pressure less than 60 mmHg. The mean heart rate was 104 beats per minute. The mean lactate and base excess were 6.8 (3.7–9.1) and minus 9 (− 14 to − 4.8), respectively. The mean temperature on presentation of the patients was 35.1 degrees Celsius. Forty-seven patients were intubated and ventilated in the ED before going to surgery. The patients spent an average of 90 min (50–200) in the emergency department before going to the operating room.

**Table 1:** Demographics, physiological parameters and injury patterns

Variable	Dead *n* = 48 *n* (%)	Alive *n* = 62 *n* (%)	*p*-value
Age in years (IQR)	34.5 (27–43)	29 (24–38)	0.10
Gender: Male	36 (43.4)	47 (56.6)	0.92
Female	12 (44.4)	15 (55.6)	
Abbreviated injury score (AIS): 2	7 (50.0)	7 (50.0)	0.44
3	7 (70.0)	3 (30.0)	
4	5 (41.7)	7 (58.3)	
5	1 (100)	0 (0.0)	
Blood pressure Mean (SD)	82.7 (45.5)	91.5 (30.7)	0.27
Inotropes (Adrenalin)	6 (66.7)	3 (33.3)	0.050*
Respiratory rate: Median (IQR)	19 (14.0–28.0)	19 (14.0–24.0)	0.89
Glasgow Coma Scale: Median (IQR)	3.0 (3.0–13.0)	9 (3.0–15.0)	0.17
Heart rate: Mean (SD)	111 (31.7)	100 (26.0)	0.070*
Temperature(°c): Median (IQR)	35.2 (33.5–36.5)	35.6 (34.9–36.3)	0.58
Lactate: Median (IQR)	8.9 (5.4–10.9)	5.4 (3.3 – 8.1)	0.002*
Base excess: Median (IQR)	− 12.1 (− 19.5–− 6.0)	− 8.0 (− 11.3–− 3.1)	0.009*
Haemoglobin: Mean (SD)	9.0 (2.6)	10.5 (2.3)	0.004*
Head injury	20 (45.4)	24 (54.6)	0.42
Thoracic injury	22 (50.0)	22 (50.0)	0.091
Abdominal injury	24 (38.1)	39 (61.9)	0.61
Long bone fractures	22 (51.2)	21 (48.8)	0.042*
Large vessel injury	8 (88.9)	1 (11.1)	0.002*
NISS score: Median (IQR)	42.0 (34.0–57.0)	35.0 (29.5–48.0)	0.10
ISS score: Median (IQR)	41.0 (30.0–51.0)	35.0 (27.0–43.0)	0.26
RTS (ED): Median (IQR)	3.8 (2.1–6.1)	5.5 (3.8–7.1)	0.003*
Red blood cells: Median (IQR)	6.0 (4.0–8.0)	3.0 (2.0–5.0)	< 0.001*
Fresh frozen plasma: Median (IQR)	4.0 (4.0–7.0)	2.5 (2.0–4.0)	< 0.001*
Platelets	17 (58.6)	12 (41.4)	0.15
Anatomy of injury			
Sacroiliac	7 (43.7)	9 (56.3)	0.99
Pubic symphysis	8 (47.1)	9 (52.9)	0.78
Acetabulum	8 (34.8)	15 (65.2)	0.34
Rami fracture	12 (33.3)	24 (66.7)	0.13
Iliac fracture	6 (28.6)	15 (71.4)	0.12
Sacral fracture	2 (22.2)	7 (77.8)	0.18
Sacroiliac & pubic symphysis	24 (55.8)	19 (44.2)	0.039*

SD (Standard deviation), IQR (interquartile range), ED (Emergency department)

Associated abdominal visceral injuries were noted in 63% of the study group, with thoracic injuries in 43.6% of cases. Head injuries were reported in 44% of the cases, twenty of which were classified as severe head injuries. Long bone fractures occurred in less than half of the group (43.9%). Seventy-four patients underwent a laparotomy. The most common solid organ injuries were the liver (*n* = 12) and spleen (*n* = 7); six patients had both spleen and liver injury. The small bowel was injured in nine patients, six colon injuries, and four had both small and colon injuries. Nine patients had major pelvic vessel injuries. External fixation was applied in 11 patients (10.6%).

The median blood products received were 4 units (2–7) of red blood cell concentrate (RBCC) 4 units (2–6) of fresh frozen plasma (FFP) and one unit pooled mega-platelets (1–4). Each pooled mega-platelet unit has between 6 and 8 units of platelets. The average cell saved blood transfused was 1000 mls (450–1750). Twenty-two patients (28.6%) patients died on the operating room table.

**Fig. 4:**
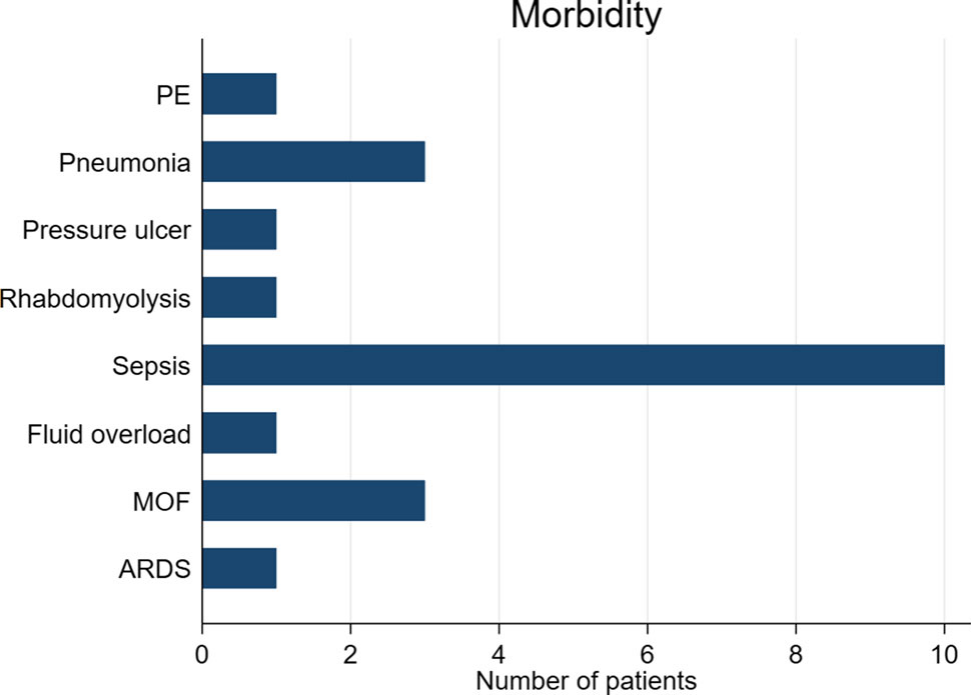
In-hospital morbidity

After preperitoneal pelvic packing, all live patients (*n* = 88) were admitted to the trauma intensive care unit (ICU). The average ICU stay was five days (0–23). The median blood products received in ICU were 2 RBCC, 2 FFP and 1 megaunit of platelets. The morbidity during hospital stays included sepsis (*n* = 10), pneumonia (*n* = 3), multiple organ failure (*n* = 3), pulmonary embolism (*n* = 10) and rhabdomyolysis (*n* = 1) (Fig. 4). None of our patients rebleed after pack removal and no one needed repacking or adjunct angioembolization in our study group.

The total in-hospital mortality rate was 43.6% (*n* = 48) in patients who underwent preperitoneal pelvic packing. The operating room table mortality was 20% (*n* = 22/110), and the mortality rate of those who survived to ICU transfer was 29.5% (*n* = 26/88). Combined sacroiliac and pubic symphysis fractures, systolic blood pressure less than 60 mmHg on presentation, the need for inotropes in the emergency department, elevated lactate and base excess and the need for massive blood transfusion were statistically associated with mortality on univariate and multivariate analysis (Table 2).

**Table 2:** Factors associated with mortality

Variable	Category	Dead *n* = 48 *n* (%)	Alive *n* = 62 *n* (%)	*p*-value
Sacroiliac & pubic symphysis	Yes	24 (55.8)	19 (44.2)	0.039*
BP	<60	16 (72.7)	6 (27.3)	0.002*
Lactate	Median (IQR)	8.9 (5.4–10.9)	5.4 (3.3–8.1)	0.002*
Base excess	Median (IQR)	− 12.1 (− 19.5–− 6.0)	− 8.0 (− 11.3–− 3.1)	0.009*
Blood	Median (IQR)	6.0 (4.0–8.0)	3.0 (2.0–5.0)	< 0.001*
Fresh frozen plasma	Median (IQR)	4.0 (4.0–7.0)	2.5 (2.0–4.0)	< 0.001*

## Discussion

Pedestrian vehicle collisions accounted for about half of the mechanism in this study group, which differs from the literature [20, 30]. This might be due to poor compliance with road safety and speeding [31, 32]. It further reflects the major mechanism associated with this mechanism of trauma. The percentage of patient undergoing pelvic packing is not high as majority of patients respond to adequate fluid and blood product resuscitation.

Early identification of a haemodynamically unstable patient with a pelvic fracture is crucial to the outcomes and intervention of the patients [31]. Our average time to surgery was 90 min, which is concerning and comparable to other studies highlighting challenges with accessing emergency theatre [32, 33]. This timing reflects the reality of the availability of emergency theatres in an overloaded trauma system, seen in low-middle-income countries. The high incidence of penetrating trauma continues to dominate our environment, and they tend to take priority over other cases [34].

The availability of angiography in other Level 1 Trauma centres has been reported to have improved outcomes in patients with haemorrhage from pelvic fracture [30, 33]. These patients may even have access to a hybrid theatre that allows the clinician to perform an operation and endovascular procedure. Preperitoneal packing may be the only option available in a resource-limited environment like ours, especially after-hours [35]. Majority of bleeds in the pelvis are from the venous plexus, and appropriate packing should be able to control most of them [20, 30, 33]. In instances where the arterial component is suspected or identified, we would directly ligate the vessel or consider a trial of internal iliac artery ligation as described by Choi et al. [36]. The bleeding in majority of patients in our patients stopped with pelvic packing.

Pelvic packing is associated with septic complications. Tötterman et al. reported up to 33% septic rates in patients who had preperitoneal pelvic packing with swabs [20, 37]. Our septic complications were lower as the packing was done under sterile conditions in the operating room, and packs were removed as soon as patients had haemodynamically normalized. There was also no repacking in our cohort. We always leave a drain in the true pelvis and monitor it closely in ICU.

Patients with pelvic fractures requiring preperitoneal pelvic packing have other associated injuries due to the high energy transfer [20]. The high ISS in or group of patients shows the patients sustained major trauma [20, 30].

Our mortality rate is comparable to that in other papers in the group of patients with life-threatening haemorrhage from pelvic fractures undergoing preperitoneal pelvic packing [21, 35]. Our patients’ haemodynamic and physiological parameters were deranged, which can explain the higher mortality rate; additionally, these patients had other associated injuries that could contribute to the overall mortality rate.

Pelvic preperitoneal packing is a lifesaving procedure in the resource-limited environment for control of pelvic haemorrhage [20, 35]. It can be used as definite management of haemorrhage or to stabilize patient for transfer to a higher-level facility or to facilitate angioembolization [35]. No patients in our study population rebled after pack removal.

## Limitations to the study

There are several limitations to the study in addition to those associated with its retrospective nature. The fluids administered prehospital were not recorded. Patients that died on the table contributed half to the overall mortality rate. The study period is over a long period with changes in some of the approach to resuscitation that may impact of current outcomes of these cases. The high associated head and chest injuries may have contributed to the overall mortality thus minimizing the impact of pelvic packing on the study group. No long-term follow-ups were done on the study to assess other complications that may be associated with the packing.

## Conclusions

Pelvic preperitoneal packing has a role in the acute management of haemodynamically abnormal patients with pelvic fractures in our environment. The associated major injuries complicate mortality outcomes in these cases. Other in-hospital complications were minimal in the study group. In the absence of immediate angioembolization, preperitoneal packing can be lifesaving. A prospective multicentre study is required to review the role of preperitoneal packing in low-middle-income environments where resources may be limited.
